# Eighteen years’ experience with tumor treating fields in the treatment of newly diagnosed glioblastoma

**DOI:** 10.3389/fonc.2022.1014455

**Published:** 2023-01-19

**Authors:** Josef Vymazal, Tomas Kazda, Tomas Novak, Petr Slanina, Jan Sroubek, Jan Klener, Tomas Hrbac, Martin Syrucek, Aaron M. Rulseh

**Affiliations:** ^1^ Department of Radiology and Department of Stereotactic and Radiation Neurosurgery, Na Homolce Hospital, Prague, Czechia; ^2^ Department of Radiation Oncology, Masaryk Memorial Cancer Institute and Faculty of Medicine, Masaryk University, Brno, Czechia; ^3^ Department of Radiation Oncology, Central Military Hospital and Faculty Hospital Motol, Prague, Czechia; ^4^ Department of Neurosurgery, Na Homolce Hospital, Prague, Czechia; ^5^ Department of Neurosurgery, Faculty Hospital Ostrava, Ostrava, Czechia; ^6^ Department of Pathology, Na Homolce Hospital, Prague, Czechia

**Keywords:** glioblastoma, magnetic resonance imaging, survival, treatment, tumor treating field (TTF) therapy

## Abstract

**Introduction:**

The prognosis of glioblastoma remains unfavorable. TTFields utilize low intensity electric fields (frequency 150–300 kHz) that disrupt cellular processes critical for cancer cell viability and tumor progression. TTFields are delivered via transducer arrays placed on the patients’ scalp. Methods: Between the years 2004 and 2022, 55 patients (20 female), aged 21.9–77.8 years (mean age 47.3±11.8 years; median 47.6 years) were treated with TTFields for newly-diagnosed GBM, and compared to 54 control patients (20 females), aged 27.0–76.7 years (mean age 51.4±12.2 years; median 51.7 years) (p=0.08). All patients underwent gross total or partial resection of GBM. One patient had biopsy only. When available, MGMT promoter methylation status and IDH mutation was detected.

**Results:**

Patients on TTFields therapy demonstrated improvements in PFS and OS relative to controls (hazard ratio: 0.64, p=0.031; and 0.61, p=0.028 respectively). TTFields average time on therapy was 74.8% (median 82%): median PFS of these patients was 19.75 months. Seven patients with TTFields usage ≤60% (23–60%, mean 46.3%, median 53%) had a median PFS of 7.95 months (p=0.0356). Control patients with no TTFields exposure had a median PFS of 12.45 months. Median OS of TTF patients was 31.67 months compared to 24.80 months for controls.

**Discussion:**

This is the most extensive study on newly-diagnosed GBM patients treated with TTFields, covering a period of 18 years at a single center and presenting not only data from clinical trials but also a group of 36 patients treated with TTFields as a part of routine clinical practice.

## Introduction

Despite considerable progress in the management of many malignant tumors, the prognosis of the most malignant primary brain tumor, glioblastoma (GBM) (1, 2), remains unfavorable. The first milestone in the management of GBM was the emergence of temozolomide (TMZ) in 1999 (3), and later its concurrent application with radiotherapy according to the Stupp protocol (4). After complex treatment, i.e., maximal debulking surgery, radiotherapy with concomitant TMZ and adjuvant TMZ chemotherapy, overall survival (OS) has increased to roughly 40–65% in the first year and 18–31% in two years (5). Few subjects survive longer than 2 years, with approximately 11% OS at 3 years, to 5% at 5 years (6–8), and less than 1% at 10 years (9).

Tumor Treating Fields (TTFields; Novocure, GmbH) may represent another milestone in the treatment of GBM. TTFields is an unconventional and unique antimitotic technique based on the principle that low-intensity, intermediate-frequency (150–300 KHz) alternating electric fields selectively kill or arrest the growth of rapidly-dividing tumor cells (10). The use of electric fields in medicine is not new; low-frequency electric fields (<1 kHz) generate action potentials in excitable cells and are used therapeutically in bone and soft tissue repair, pain control and stimulation in neurology and cardiology. High frequency (>10 MHz) alternating fields generate heat in tissues by dielectric losses. Their therapeutic applications include thermoablation, diathermy and hyperthermia (11). The antimitotic effect of intermediate-frequency TTFields is due to interference with the proper formation of the mitotic spindle during metaphase and anaphase and by dielectrophoresis of macromolecules and organelles during cytokinesis. This effect has been proven both *in vitro* and *in vivo* (10). TTFields also have the potential to be used outside the brain; current Novocure sponsored TTFields clinical trials are focused on pancreatic (NCT03377491), and lung (NCT04892472) cancers (12–14), and the FDA has recently approved the use of TTFields in mesothelioma (14).

Currently, more than 1,300 certified centers prescribe TTFields for GBM therapy in the U.S. and there are also many centers in the EU, Switzerland, Japan, Israel and other countries. In many countries, the treatment is covered by health insurance, while in several other countries the issue is still being evaluated by regulatory authorities. Most studies focusing on the use TTFields in GBM have been based on the results of clinical trials. As the population of patients enrolled into clinical trials and those in routine clinical practice may differ (15), it is desirable to also report results of patients treated outside clinical trials (16). Furthermore, there is a lack of studies describing patient outcomes following TTFields therapy with a long follow-up duration. Thus, the aim of the present study was to describe outcomes of TTFields therapy for a consecutive cohort of newly-diagnosed glioblastoma patients treated both within the clinical trial as well as routine clinical practice settings over a period of 18 years.

## Material and methods

### Patient characteristics

All patients provided written, informed consent for clinical trial participation or treatment within the clinical practice setting, including consent for surgery, MRI and follow-up examinations. All patients provided written, informed consent regarding the publication of their anonymized data for scientific purposes.

Between August 2004 and August 2021, 55 patients (20 female, median age at diagnosis 47.6 years, SD ± 11.8 years [range 21.9–77.8 years]) started treatment with TTFields for newly-diagnosed and histologically-confirmed supratentorial GBM at our center (Na Homolce Hospital in Prague). According to current terminology, patients with astrocytoma grade IV were also included. Patients with recurrent disease were not included. Eleven patients (20%) were treated between 2004–2006 in the pilot EF07 trial, 8 patients (15%) as a part of the EF14 study, and 36 patients (65%) in the routine clinical setting. All patients had a Karnofsky performance score of 70 or more at the beginning of TTFields therapy. Patient and diagnostic characteristics are summarized in **Table 1**.

**Table 1:** Patient characteristics.

Characteristic	TTFields patients N=55	Control patients N=54	P value
Age			
Median (range)	47.6 (21.9-77.8)	51.7 (27.0-76.7)	0.08
Mean, SD	47.3 ± 11.8	51.4 ± 12.2	0.08
**Female**	20 (36%)	20 (37%)	
**Median follow-up (months)**	24.3 (8.0-210)	23.1 (6.9-172)	0.44
Resection status			
Gross total	38	43	
Subtotal/partial	17	10	
Biopsy	0	1	
IDH status			
wild type	23	23	
mutated	4	1	
unknown	28	30	
MGMT promotor status			
methylated	15	9	
unmethylated	7	8	
Unknown	31	35	
Resection side			
Left hemisphere	19	23	
Right hemisphere	36	28	
Bilateral	0	3	
Resection region			
Frontal	12	18	
Frontotemporal	2	3	
Frontoparietal	1	2	
Parietal	11	7	
Parieto-occipital	2	5	
Temporal	17	14	
Temporoparietal	5	3	
Temporo-occipital	0	1	
Occipital	5	0	
Midline	0	1	
Karnofsky performance score			
Median	80	80	
Range	70–100	60–100	
90-100	26	24	
80	24	25	
70	5	4	
60	0	1	
Postoperative volume in PR			
Median	1.017	0.496	
Mean	1.841	0.657	
SD	2.669	0.507	

PR, partial resection.

After a thorough search of our hospital database, 104 consecutive patients treated in the same period for newly-diagnosed, histologically-confirmed supratentorial GBM were selected. In 60 of these control patients, we were able to obtain all necessary information for the present study: clinical status, histology, MRI examination, the date of progression, and the date of death. Six patients were further excluded due to early GBM progression (less than 4 months). This interval was chosen according to the median time between surgery and TTFields initiation in those patients treated by TTFields. Thus, the only selection criteria for control patients were data completeness, progression-free survival more than 4 months after surgery and Karnofsky performance score of 70 or more. Thus, 54 control patients were included (20 female, median age at diagnosis 51.7 years, SD ± 12.2 years [range 27.0–76.7 years]). Data collection was closed on June 15, 2022.

### Missing data

There were no missing data regarding survival. Censored data in the present study indicates that the patient either did not progress (PFS) or is still alive (OS). All patients lost from evidence were excluded. In four control patients, it was not possible to unequivocally assess the progression date, however, the date of death was available. Therefore, the PFS group is reduced by 4 patients in comparison to the OS group.

### MRI and clinical protocols

MR imaging with the administration of a gadolinium-based contrast agent was performed at 1 Tesla, 1.5 Tesla and 3 Tesla magnetic field strengths. Patients from the EF07 study were scanned at 1 Tesla and three surviving patients were followed at 1.5 Tesla or 3 Tesla after 2009. Patients from the EF14 study were followed at 1.5 Tesla and those from the clinical TTFields group at 3 Tesla. Progression was confirmed using MacDonald and later RANO criteria (17, 18).

### Histology and integral diagnosis

All patients had grade IV glioblastoma according to diagnostic criteria at the time of diagnosis. Molecular-biological data, particularly (O6-methylguanine-DNA-methyltransferase) MGMT promotor methylation and (isocitrate dehydrogenase) IDH mutation, were not available in all patients as this information was not routinely collected at that time. The available data are summarized in **Table 1**. Nevertheless, the number of known IDH wild type patients were the same in both groups (23 patients). In patients treated by TTFields, there were 4 patients with positive IDH mutation compared to 1 patient in the control group. The number of subjects with unknown IDH status was similar in both groups (28 versus 30). Similarly, the number of patients with unknown MGMT methylation status was comparable in both groups (31 versus 35 patients). Fifteen patients treated by TTFields and 9 patients from the control group had methylated MGMT promotor status, while seven patients treated by TTFields and eight patients from the control group had unmethylated MGMT promotor status.

### TTFields therapy and usage

TTFields treatment was delivered through four transducer arrays with nine insulated electrodes placed on the shaved scalp of the patient. This basic concept did not change throughout the study period; between the years 2004 and 2022. A portable device generating the tumor-treating alternating field of 200 KHz frequency became more patient-friendly over time. All treatment was done on an outpatient basis. The patients and their families or caregivers were trained to operate the device independently. (8). The compliance of TTFields treatment was followed in each patient monthly.

## Results

### Basic treatment characteristics

All patients treated with TTFields and all patients in the control group underwent standard treatment for GBM: gross total or subtotal/partial resection of the tumor (only 1 patient with biopsy was included), followed by radiotherapy with concomitant TMZ. Patients treated after 2005 received therapy according to the Stupp protocol. Adjuvant cycles of TMZ followed in both groups of patients; patients treated with TTFields as well as patients in the control group. There were no significant differences in therapeutic strategy between both groups. Patients treated before 2005 also received radiotherapy with concomitant temozolomide, followed by adjuvant temozolomide. This therapy was often used in clinical practice before the publication of the Stupp protocol (4).

### TTFields therapy and usage

The median interval between surgery (i.e., the time of diagnosis) and TTFields initiation was 3.8 months in the EF14 study (8) and 4.38 months in patients treated as a part of routine clinical practice. No serious adverse events related to TTFields therapy were recorded. The most frequent adverse event was skin irritation that usually responded to local corticosteroid application. The compliance of 36 clinical patients was mean 74.8% (median 82%; percentage of day treatment applied). The median PFS of these patients was 18.16 months. Seven patients with compliance ≤60% (mean 46.3%, median 53%, range 23–60%) had significantly shorter median PFS of 7.95 months (p=0.0356).

### MRI and clinical examinations

All patients underwent regular MRI examinations, as well as clinical evaluation by a board-certified neurologist or neurosurgeon. In the EF07 study, the interval between MRI and clinical evaluation was 1 month, in the EF14 study 2 months, and in clinical patients 2–3 months. In the control group, the interval between examinations was approximately 3 months. MRI examinations were performed with a standard protocol and with the administration of an intravenous gadolinium-based contrast agent.

**Figure 1:**
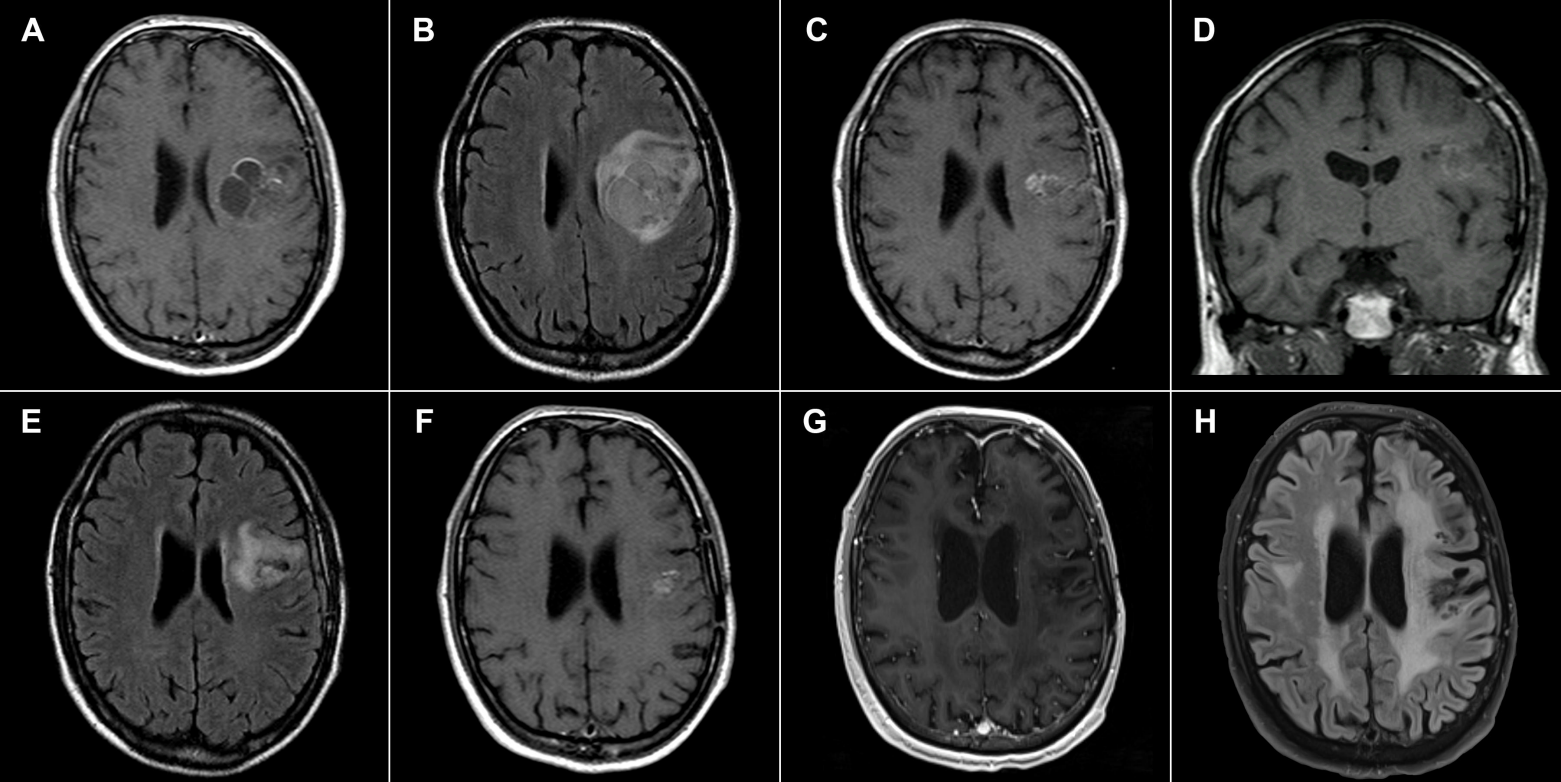
43-year-old male. **(A)** January 2004, 1 Tesla scanner: T1-weighted postcontrast spin echo preoperative image showing a GBM in the left central region. **(B)** Corresponding FLAIR image. **(C)** July 2004, 1 Tesla scanner: T1-weighted postcontrast spin echo postoperative image with a residual enhancing tumor after a partial resection **(D)** T1-weighted pre-contrast spin echo image from the same examination with minimal hyperintense signal at the border of the lesion **(E)** Corresponding FLAIR image. **(F)** August 2005 1 Tesla scanner: T1-weighted postcontrast spin echo image showing a decrease of the postcontrast enhancement suggesting a residual tumor regression. **(G)** May 2022, 3 Tesla scanner: T1-weighted postcontrast MP-RAGE sequence 217 months after surgery with no signs of residual tumor or its recurrence showing dystrophic and atrophic changes **(H)** Corresponding FLAIR image with dystrophic and post-radiation changes.

**Figure 2:**
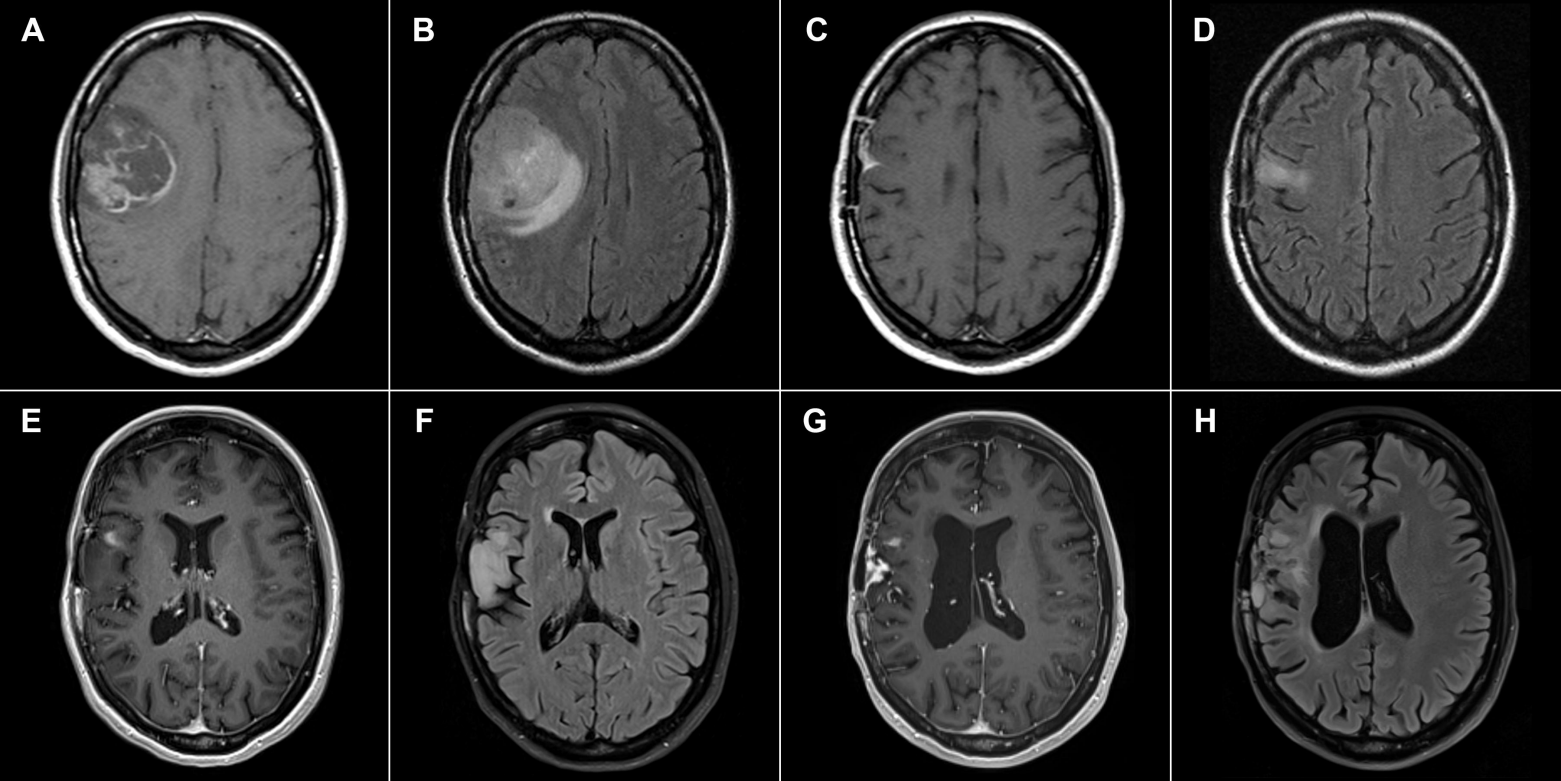
33-year-old female. **(A)** November 2005, 1 Tesla scanner: T1-weighted postcontrast spin echo preoperative image showing a GBM in the right central region. **(B)** Corresponding FLAIR image. **(C)** April 2006, 1 Tesla scanner, T1-weighted postcontrast spin echo postoperative image with a residual extra-axial enhancement. No enhancing residual tumor is present. **(D)** Corresponding FLAIR image. **(E)** 3 Tesla scanner, January 2021, T1-weighted postcontrast MP-RAGE sequence 181 months after surgery with signs of tumor recurrence in the right opercular region. **(F)** Corresponding FLAIR image. **(G)** 3 Tesla scanner, May 2022, T1-weighted postcontrast MP-RAGE sequence with a second recurrence in the same region. **(H)** Corresponding FLAIR image.

Patients in the EF07 trial were scanned every month during the trial; two of three surviving patients are currently examined annually. **Figure 1** shows preoperative and postoperative MRI images of a GBM patient with no recurrence 217 months after surgery, who underwent TTFields therapy for one year. The patient has slight residual dysphasia and is able to lead an independent life including work; the Karnofsky performance score is 90. **Figure 2** shows preoperative and postoperative MRI images of a patient with recurrence 181 months after surgery. This patient is currently undergoing a second application of TTFields therapy with minimal left hemiparesis, leading an independent life, with a Karnofsky scale score of 90. The second recurrence was detected 196 months after surgery in the same region and was treated using radiosurgery.

**Figure 3:**
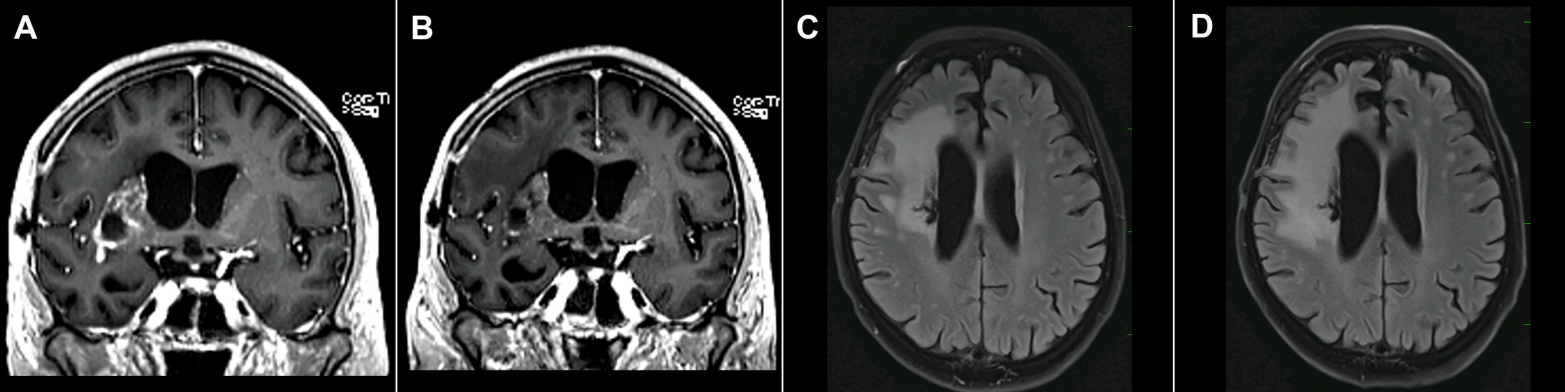
59-year-old female. **(A)** April 2019, 3 Tesla scanner “bevacizumab-like” TTFields effect: T1-weighted postcontrast gradient echo postoperative image with enhancing residual GBM in the region of the basal ganglia on the right side. **(B)** July 2019, 3 Tesla scanner: T1-weighted postcontrast gradient echo image (identical scanner, sequence parameters, type and dose of the contrast agent [Gadovist 6 ml]) shows a decrease in postcontrast enhancement. However, the neurological status of the patient progressed. **(C)** April 2019, 3 Tesla scanner, FLAIR image and **(D)** July 2019, 3 Tesla FLAIR image show edema progression at the time when enhancement decreased.

In some TTFields patients, an “bevacizumab-like” effect was observed on follow-up MRI (**Figure 3**). While postcontrast enhancement decreased, the neurological status of the patient progressed.

**Figure 4:**
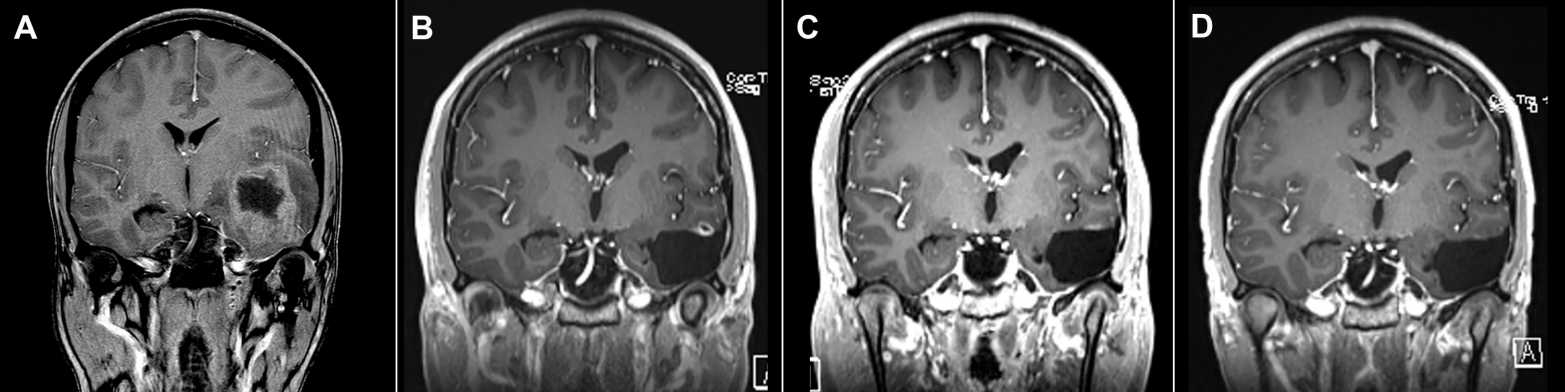
26-year-old female **(A)** November 2017, 1.5 Tesla scanner: T1-weighted postcontrast gradient echo preoperative image showing GBM in the left temporal region. **(B)** April 2019, T1-weighted postcontrast gradient echo image showing long-lasting postoperative enhancement suspicious of residual tumor. **(C)** August 2019, T1-weighted postcontrast gradient echo image reveals a decrease in enhancement. **(D)** March 2022, T1-weighted postcontrast gradient echo image with no signs of tumor recurrence.

In some patients, it was difficult to differentiate between small residual or recurrent tumor and late residual post-irradiative changes. **Figure 4** shows a 30-year-old female on TTFields therapy with a small, residual enhancing area 16 months after gross total resection of (IDH mutant) GBM. The area of enhancement regressed 20 months after surgery and the patient currently shows no signs of recurrence 51 months after resection.

### PFS and OS

**Figure 5:**
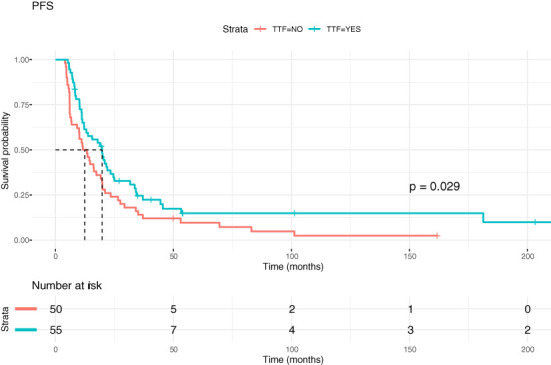
Kaplan-Meier curves showing progression-free survival (PFS) in GBM patients with and without TTFields therapy.

**Figure 6:**
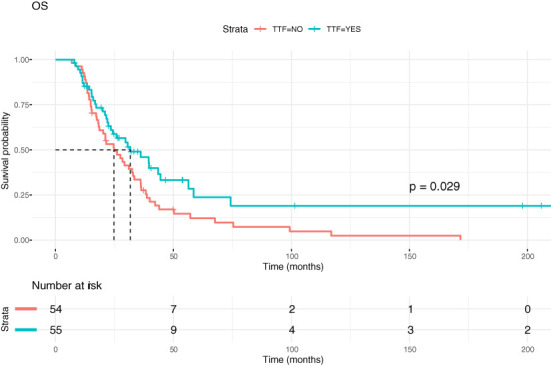
Kaplan-Meier curves showing overall survival (OS) in GBM patients with and without TTFields therapy.

**Figures 5**, **6** show Kaplan-Meier (KM) estimation of PFS and OS for patients treated with and without TTFields. The dates of progression and/or death were available in all patients in both groups except for four patients from the control group (PFS), which were not included in the KM analyses; OS in these patients was available.

Median PFS for patients within the TTFields group was 19.75 months, while median PFS for patients without TTFields treatment was 12.45 months, p=0.031). Corresponding values for median OS were 31.67 months versus 24.80 months (p=0.028). The hazard ratio for PFS was 0.64 and for OS 0.61. **Table 2** summarizes the one-year, two-year and five-year survival of TTFields patients and controls as well as other survival data.

**Table 2:** Survival data.

Survival (months; 95% CI)	TTFields patients N=55	Control patients N=54	P value
Overall survival			
Median	31.67	24.80	
1-year	0.87 (0.79-0.96)	0.93* (0.86-0.99)	
2-year	0.61 (0.49-0.76)	0.53 (0.41-0.68)	
5-year	0.24 (0.12 – 0.45)	0.12 (0.06 – 0.26)	
HR	0.61		0.028
Progression free survival			
Median	19.75	12.45	
1-year	0.65 (0.54– 0.79)	0.50 (0.38 – 0.66)	
2-year	0.37 (0.30 – 0.58)	0.24 (0.14 – 0.39)	
5-year	0.15 (0.07 – 0.30)	0.1 (0.04 – 0.23)	
HR	0.64		0.031

*This value may be biased by eliminating 6 patients with early progression.

## Discussion

TTFields is a novel antimitotic technique approved in many countries for the treatment of both primary and recurrent GBM (19). Its methodology is based on the interference of alternating electric current (AEC) with the cell cycle. Although the fields created by an AEC have been utilized in clinical medicine for years, the antimitotic effect of AEC is a novel approach in oncology, far different from standard chemo- and radiotherapy methods. Thus, it is justifiable that TTFields, similar to any other new technique and method in clinical medicine, requires awareness in terms of its effectivity and routine clinical use. Indeed, after publishing multicentric clinical studies with positive results (8, 10, 20), some neurooncologists called for prudence in accepting this technique (16), and asked for more data based not only on clinical trials but also on routine clinical practice (19, 20).

The current study thus presents not only data from clinical trials but also a group of 36 patients treated with TTFields as a part of routine clinical practice. Covering a period of TTFields therapy between 2004–2022, our data represent the most extensive experience with this technique in newly-diagnosed GBM patients, with three patients surviving from the first pilot EF07 trial performed between 2004–2006. Our results demonstrate a positive and statistically significant effect of TTFields therapy on both PFS and OS in patients with newly-diagnosed GBM, in comparison with a control group treated over the same period of time and at the same institution. Survival statistics of newly-diagnosed GBM patients with a standard treatment regimen without TTFields differ, although a trend toward longer survival is logically plausible. Raj et al. published a nationwide study from Finland (21), where the one-year survival of newly-diagnosed GBM patients increased from 34% to 43% between the periods of 2007–2013 and 2000–2006, respectively. A similar increase in one-year survival with similar values was also reported from the USA (2). Conversely, in 2017 Kelly et al. (22) estimated one-year survival at 67%, very close to the 65% published by Stupp et al. (8).

In the study by Stupp et al. (8) on newly-diagnosed GBM patients in the EF14 study, the authors reported two-year survival of TTFields patients at 43% (31% for controls), and five-year survival of 13% for TTFields patients (5% for controls). Our data show better results for both groups: two-year survival in the TTFields group was 60% and 53% in the control group, with five-year survival of 24% for TTFields patients and 12% for controls. There may be several reasons for these differences. First, we excluded six patients from the control group that progressed within the first 4 months after surgery (all of whom did not survive beyond the first year). This interval was chosen according to the median time between surgery and TTFields initiation in patients treated by TTFields (16), as patients that progress very early after surgery will not become candidates for TTFields therapy. Additionally, the standard of care (including supportive care) has likely improved since the EF14 study was conducted. Although our results were superior to than those of EF14 with respect to PFS and OS, it is interesting to note that OS was very close in both groups the first year after diagnosis, and even somewhat higher in the control group (87% versus 90%). Both values are very high and the control group is biased by the exclusion of patients with very early progression.

Another opinion challenging TTFields therapy is that, for ethical reasons, there were no sham control patients in any TTFields trial (16, 19). The principle of TTFields makes a sham device nearly impossible to use, as there is a high probability that the patient would be able to distinguish between a real and sham device. A different approach is the evaluation of subjects according to treatment compliance, as patients in the EF14 trial showed a dependence of PFS and OS on TTFields usage (23, 24). Thus, we selected patients with lower treatment usage (≤60%) and compared their PFS with control patients as well as with patients with usage >60%. A significant difference was found in PFS between the patients with high and low TTFields usage, and no statistical difference between the patients with low usage and controls. All patients treated with TTFields at our department have thorough documentation, thus a selection criterion for control patients was a similar level of documentation including all necessary data to determine the date of progression and known date of death. Therefore, in our KM plots there are no censored patients that were lost to follow up; censored data correspond to patients that are still alive and/or without progression.

TTFields patients underwent MRI examinations more frequently in comparison to controls, thus PFS may theoretically be shorter in TTFields patients compared to controls due to greater precision. PFS may be by this mechanism somewhat biased because the recurrence is detected earlier in comparison with the control group. From a neuroradiological point of view, it is critical to emphasize the importance of the use of the same field strength, identical imaging sequences, and also (due to the fact that MRI contrast agents differ in their relaxivity (25) the use of the same contrast agent and relative dose in follow up exams. In this way, with the help of advanced MRI techniques such as MR spectroscopy (26), it is possible to detect tumor recurrence earlier and in a reliable way.

A long period of patient recruitment and follow-up, reaching almost two decades, is a strength of this study, but also a weakness due to possible changes in therapy or supportive care over this long period. The main difference may be in the gradually evolving techniques of postoperative radiotherapy from 3D-conformal techniques to intensity-modulated beam radiotherapy (including volumetric modulated arc therapy). It should be mentioned that no study to date has confirmed the superiority of one radiotherapy technique. As the same technological developments were also reflected in the control group of patients, we do not expect that treatment changes over the study period of two decades significantly affect the comparison of outcomes.

In conclusion, this is the most extensive study of newly-diagnosed GBM patients treated with TTFields, covering a period of 18 years at a single center in patients that participated in clinical trials, as well as in patients treated as a part of routine clinical practice. Our study demonstrates the positive effect of TTFields on both PFS and OS in patients with newly-diagnosed GBM. Three patients have been followed for 16–18 years after initial surgery; two with no recurrence and one with recurrence after 15 years.

## Data availability statement

The raw data supporting the conclusions of this article will be made available by the authors, without undue reservation.

## Ethics statement

The studies involving human participants were reviewed and approved by Ethics Committee Na Homolce Hospital, Prague, Czechia. The patients/participants provided their written informed consent to participate in this study.

## Author contributions

JV and AR designed the study, performed clinical and imaging controls, analyzed and interpreted the data and writing of the manuscript. TK designed the study, performed clinical controls, analyzed the data and contributed to the writing of the manuscript. TN, JS, JK, TH performed clinical controls and contributed to the writing of the manuscript. PS analyzed the data, performed technical controls and contributed to the writing of the manuscript. MS analyzed the data, performed histological verification and contributed to the writing of the manuscript. All authors contributed to the article and approved the submitted version.
